# Behavior of Wear Debris and Its Action Mechanism on the Tribological Properties of Medium-Carbon Steel with Magnetic Field

**DOI:** 10.3390/ma12010045

**Published:** 2018-12-24

**Authors:** Hongxin Shi, Sanming Du, Chao Sun, Chenfei Song, Zhenghai Yang, Yongzhen Zhang

**Affiliations:** National United Engineering Laboratory for Advanced Bearing Tribology, Henan University of Science and Technology, Henan Province, Luoyang 471000, China; hyshhongxin@163.com (H.S.); dsming_001@163.com (S.D.); cfsong@haust.edu.cn (C.S.); yzh772029@163.com (Z.Y.)

**Keywords:** magnetic field, medium-carbon steel, wear debris, wear, mechanism

## Abstract

Friction tests were conducted on self-matched pairs of medium-carbon steel using a pin-disk tribometer in an ambient laboratory environment with and without wear-debris removal, in order to clarify the influence of wear debris on the tribological properties of steels that were exposed to magnetic fields. The wear debris and worn surface were observed and analyzed. In the case that the wear debris was removed, the vast majority of wear debris was large, scarce oxidation, and no agglomeration, the grooves of various shapes and discontinuities, and no oxide layer were formed on the worn surface, severe wear occurred throughout the friction process. When the wear debris was not removed, the wear debris became fine, agglomeration and oxidation, a debris layer was formed on the worn surface, and the wear mode transitioned from severe to mild occurred during friction process. The results reveal that the re-entering of wear debris into the friction area is essential for the formation of a wear-debris layer and that an anti-wear effect can be achieved via the wear-debris layer formed on the worn surface during the friction process with a magnetic field.

## 1. Introduction

Some constitutive components in mechanical and electronic equipment (e.g., electromagnetic brake, linear motors, and power motors, etc.) operate in the presence of magnetic fields with the development and application of electromagnetic technologies [1]. The behavior of paramagnetic and ferromagnetic materials is influenced when they are exposed to magnetic fields. During friction processes under the action of a magnetic field, oxygen in the ambient environment can be attracted by the magnetic field, which leads to an increase in the local oxygen partial pressure around the couple [2]. On the other hand, the ferromagnetic wear debris can also be absorbed on the worn surface [3]. Therefore, the tribological properties of ferromagnetic materials are affected by the applied magnetic field [4,5,6,7,8].

In order to make better use of the magnetic field during friction, the tribological mechanism of metal materials that are exposed to magnetic fields has been studied. Generally speaking, oxidation was thought to be one of the factors affecting the friction and wear properties of materials [9]. In comparison with the traditional friction without magnetic field, the effect of oxidation on the tribological properties of materials exposed to magnetic field is more significant [10,11,12,13]. Further, wear debris is also assumed to influence the tribological properties of materials subjected to a magnetic field [1,14,15,16]. However, in studies on the oxidation mechanism during friction, wear debris is unavoidable [17,18,19,20]. Therefore, it plays an important role in dictating tribological properties during friction under the action of a magnetic field. 

To analyze the role of wear debris in the friction process, friction tests between couples of metal materials were conducted without and with a magnetic field. The function of wear debris was investigated by comparing the two test results [21,22]. Here, it was supposed that the wear debris could not be attracted onto the worn surface of the specimen when there was no magnetic field. Nevertheless, magnetization of the specimens can occur when ferromagnetic materials are rubbed against each other [23,24]. This results in some of the wear debris being attached on the surface even when no magnetic field is employed during the friction process. Therefore, the behavior and effect of wear debris in the presence of a magnetic field should be further investigated by taking special measures during the friction process.

With this aim, friction tests were conducted in this study on medium-carbon steel self-matched pairs using a pin and disk friction test machine either equipped with or without a wear-debris removal device. Medium-carbon steel belongs to ferromagnetic material and its tribological properties are remarkably influenced by a magnetic field [15]. Therefore, it is more favorable to investigate the effect mechanism of a magnetic field on the tribological properties of ferromagnetic materials to adopt of medium-carbon steel as friction pair material. Two types of wear-debris removal methods, irreversible and reversible, have been reported in previous studies. Some examples of irreversible methods are as follows. Micro-pores with diameters in the range of 100–120 µm were machined on the specimen surface to allow the escape of wear debris into there in the process of friction [25]; a groove-textured surface can remove wear debris from the friction interface into the grooves [26,27]; and, a radial grooved disk was used to throw wear debris away when it was rotating [28]. In the case of reversible methods, brushes, blowing air, and magnetic fields were employed at the contact interface to clear wear debris [29,30].

The above-described methods achieved the desired result of wear-debris removal from the specimen surface during the friction process in the absence of a magnetic field. However, when a magnetic field is present, there occur several problems. Firstly, micro-pores and grooves are easy to fill up and the effect of debris removal is weakened because a large number of wear debris particles are absorbed on the worn surface in the presence of a magnetic field. Secondly, it is impossible to eliminate wear debris completely in the case of radial grooved disks; further, wear debris cannot be completely cleared using a brush, as it is firmly adsorbed on the worn surface under the action of a magnetic field. Third, blowing air flings around the wear debris during friction, which contaminates the surroundings. Fourth, mutual interference can occur between the magnetic field that was used to remove wear debris and the magnetic field acting as an external factor in the friction process. Lastly, the surface or structure of the friction specimen might undergo changes and these cannot be recovered in the case of irreversible methods.

Therefore, irreversible and reversible methods are not suitable for removing wear debris during the magnetized friction of ferromagnetic materials. Combined with the characteristics of the wear debris during a magnetization friction process, a suction device was employed for removing wear debris; it can overcome the disadvantages that are faced by irreversible and reversible methods. Thus far, there have been very few investigations on the behavior and effect of wear debris during friction in the presence of a magnetic field after removing wear debris from the worn surface. In this study, the behavior of wear debris and its effects on the tribological properties were analyzed via by comparing the friction results obtained with and without using the wear-debris removal device. Henceforth, the removal device is referred to as RD.

## 2. Experimental Methodology

The material of the pin and disk used in this study was as-normalized medium-carbon steel, whose nominal chemical composition (wt.%) was 0.44 C, 0.60 Mn, 0.25 Si, 0.04 P, 0.04 S, and balance Fe. The diameter and length of pin specimens are 5 mm and 40 mm, respectively. The size of disc specimen is 5 mm in diameter and 40 mm in thickness. The pre-friction surfaces of pin and disk specimen were processed to attain the roughness of 0.8 and 1.6, respectively. The rotating radius of relative motion of the pin on the disk was 72.5 mm. 

**Figure 1:**
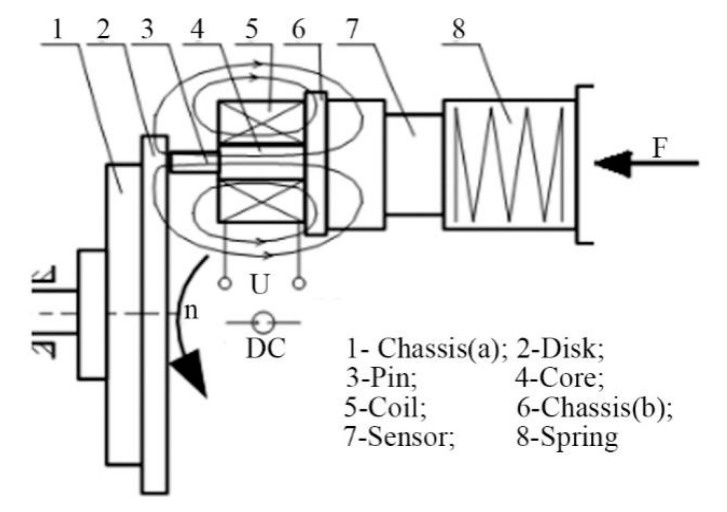
Schematic diagram of the tribometer.

Friction tests were carried out using a pin-on-disk type tribometer at room temperature in an ambient atmosphere. Figure 1 shows a schematic diagram of the tribometer. The disk specimen is rotated by an electric motor with a rotating speed *n* in the vertical plane and the pin specimen is fixed; under such conditions, sliding friction tests are carried out. A magnetic field perpendicular to the sliding surface can be achieved via current flowing in a coil. In this study, direct current (DC) flows through the coil and it produces a steady DC magnetic field. The RD was placed on the pin-on-disk type tribometer during the test, as described below.

Keeping the sliding speed, load, and friction time fixed at 0.54 m/s, 100 N, and 1800 s, respectively, the magnetic-field intensity (*H*) was varied (0, 2.1 × 10^3^, 4.3 × 10^3^, 8.7 × 10^3^, 17.4 × 10^3^, 26.1 × 10^3^, 43.5 × 10^3^, and 60.9 × 10^3^ A/m). Before the friction tests, the specimens and components for fixing the specimens were demagnetized using a demagnetizing device (WLM-TB60). The pin and disk specimen surfaces were polished using a 1000# abrasive paper to remove any foreign matter on the surface of the specimen. Later, the specimens were cleaned with acetone, dried naturally, and installed on the tribometer.

The weight of the pin specimen before and after friction was measured using an electronic balance up to a precision level of 0.1 mg. The wear coefficient, *K_p_*′ (mm^3^/Nm), was calculated using the following equation.

(1) $$K_{p}\prime =(W_{a}-W_{b})/(\rho vtP)$$

Here, *W_a_* and *W_b_* are the weights of the pin specimen before and after the friction test, respectively. *ρ* represents the density of the pin specimen material (g/mm^3^), *v* is the friction speed (m/s), *t* is the friction time (s), and *P* is the actual vertical load (N).

The dynamic friction coefficient and pin displacement in the friction process were processed and analyzed after the friction tests. The worn surfaces and cross sections of the pin specimen and wear debris were observed and analyzed using a scanning electron microscope (SEM) equipped with an energy spectrum analyzer (EDS). X-ray diffraction (XRD) analysis was conducted to determine the phases in the pin specimen adjacent to the worn surface. 

## 3. Location Determination of the Wear-Debris Removal Device (RD)

**Figure 2:**
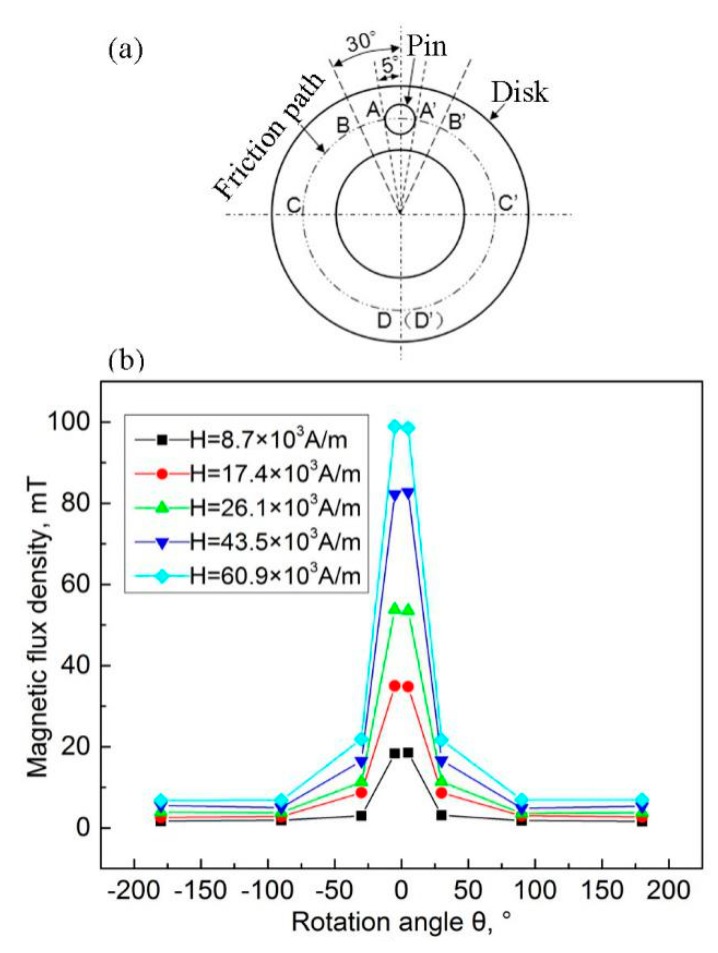
Measurement of magnetic induction intensity. (**a**) Measured location and (**b**) measured values.

In this study, the suction inlet location of the RD onto the disk was mainly determined by the attractive forces between the wear debris and the worn surface of the disk specimen. In order to analyze the attractive force between the wear debris and the worn surface of the disk specimen, the magnetic induction intensity of the worn surface of the disk specimen was measured using a gauss meter (HT201) at A, A’, B, B’, C, C’, D, and D’, as shown in Figure 2a.

**Figure 3:**
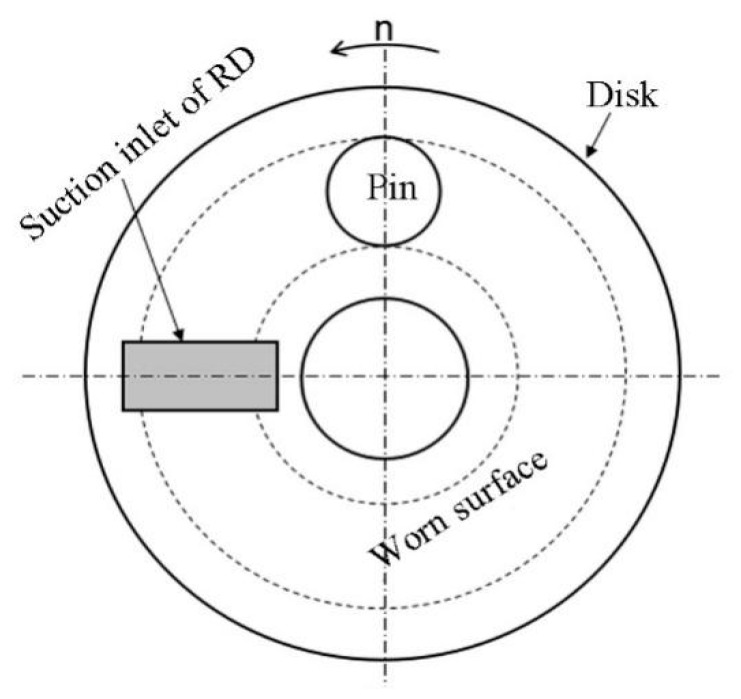
Suction inlet location of the removal device (RD).

The magnetic induction intensity values measured on the surface of the disk specimen when there was no gap between the pin and disk are shown Figure 2b. It can be seen that the distribution of magnetic induction intensity on the surface of the disk specimen at various magnetic field intensities was almost constant. The intensity gradually reduced with an increase in the corresponding angle and it reached a stable value when the angle was greater than ±90°. In this case, the stable magnetic induction intensity varied from 1.8 mT to 6.9 mT with an increase in the magnetic-field intensity. According to the Maxwell formula, the magnetic attraction force between two objects increases with an increase in magnetic induction intensity, as described below. Therefore, the suction inlet of the RD was located at an angle of 90° with respect to the friction path of the disk specimen, as shown in Figure 3. 

When the gap between two objects is close to zero, the Maxwell formula (Fd=B2S2μ0) can be used to calculate the magnetic attraction force. In the formula, *F_d_* refers to the magnetic attraction force between the two objects, *B* is the magnetic induction intensity, *S* is contact area between the two objects, and *μ*_0_ is the permeability of vacuum. Supposing that the specification of the wear debris is 10 μm × 10 μm × 10 μm, *F_d_* can be calculated to be 1.9 × 10^–9^ N, when the corresponding angle is ±90°. In order to wipe off the wear debris at this location, the force that is applied on the wear debris should be more than *F_d_* and its direction should be opposite to that of *F_d_*. 

The suction inlet of the RD that was used in this study is shaped like a rectangle with dimensions of 10 mm × 20 mm; the aim is to cover the worn path in the width direction as shown in Figure 3, which guarantees wear-debris removal from the worn surface of the disk specimen. A negative pressure of ~30 kPa comes into being where the wear debris is removed, owing to the rotation of the centrifugal blower in the RD. An attractive force of 6 N is generated for the wear debris. As this value is much larger than *F_d_*, wear debris on the worn surface of the disk specimen can be removed.

**Figure 4:**
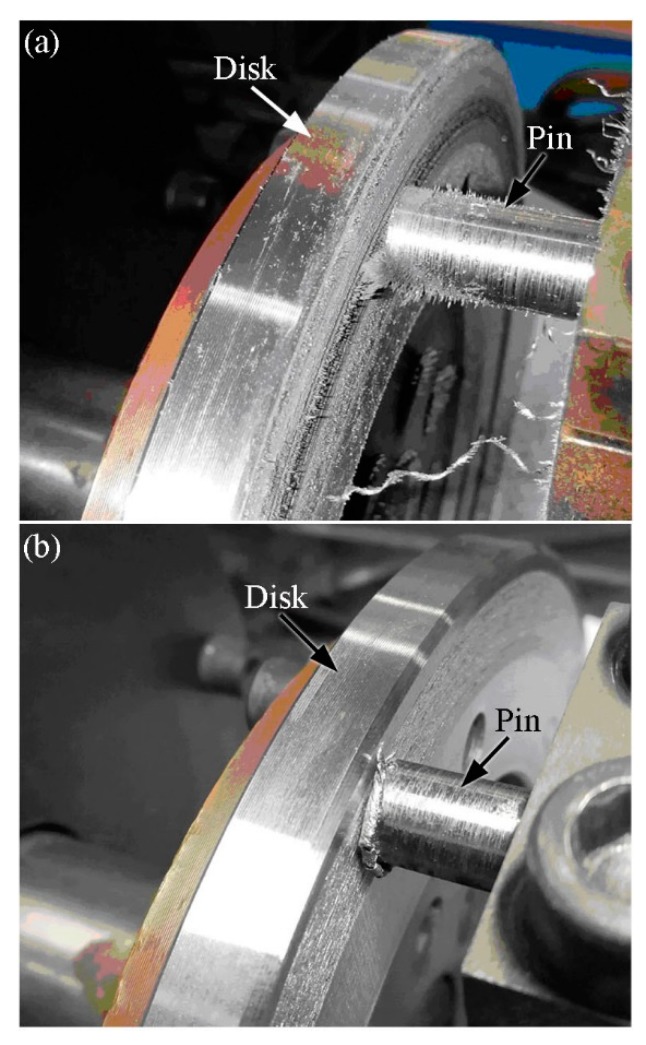
Appearance of the friction couple. (**a**) Without using RD and (**b**) using RD.

Figure 4a,b depict the appearance of a friction couple subjected and not subjected to RD action during the friction process, respectively. As shown in Figure 4a, a large proportion of the wear debris was absorbed on the worn surface of the disk specimen when the RD was not used. However, little wear debris was observed when the RD was used, as shown in Figure 4b. Therefore, wear debris on the worn surface of the disk specimen can be fully removed using the RD.

## 4. Experimental Results and Discussion

### 4.1. Features of the Wear Debris

**Figure 5:**
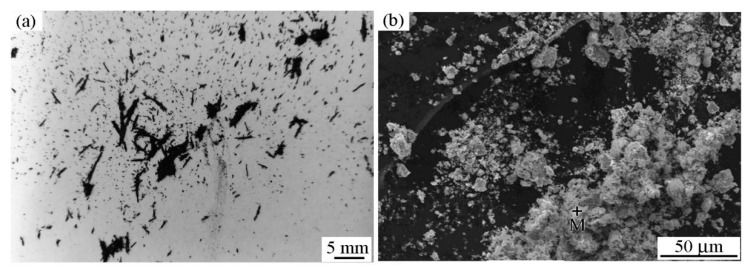
Morphology of wear debris when the RD was not used; *H* = 26.1 × 10^3^ A/m. (**a**) Macro-photograph and (**b**) scanning electron microscope (SEM) image.

Figure 5a,b show a macro-photograph and SEM image of the wear debris that were collected in the friction process, respectively, when the RD was not used. As shown in Figure 5a, agglomeration was observed in the wear debris. This indicates that magnetization of the wear debris occurred. In addition, the wear debris was black in this case. The size of the wear-debris particles was small, even reaching the micron level, as shown in Figure 5b. The EDS results in zone M (shown in Figure 5b) are listed in Table 1. The oxygen content of the wear debris was high, which might be qualitatively considered to be due to the oxidation of wear debris during the friction process. This is also the reason why the wear debris was black.

**Figure 6:**
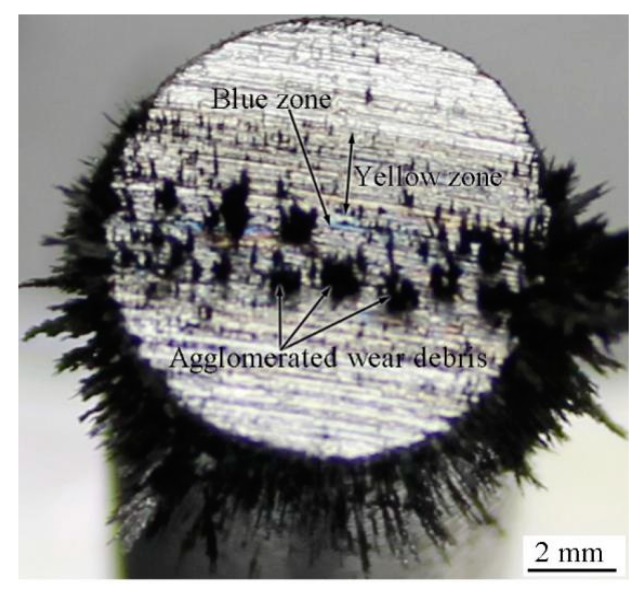
Macro-photograph of the pin when the RD was not used. *H* = 26.1 × 10^3^ A/m.

The agglomeration of wear debris can be attributed to magnetization and refinement; further, the wear-debris particles attract one another due to the action of the magnetic field during the friction process. The extent of agglomeration increased with an increase of the magnetic-field intensity and decrease in wear-debris particle size. Figure 6 shows a macro-photograph of the pin specimen at a friction time of 150 s. As shown, an agglomeration of wear debris on the frictional area of the pin can be observed. Therefore, agglomeration occurred not only in the wear debris detached from the specimen, but also in that on the worn surface and the frictional area, as shown in Figure 4a and Figure 6.

**Figure 7:**
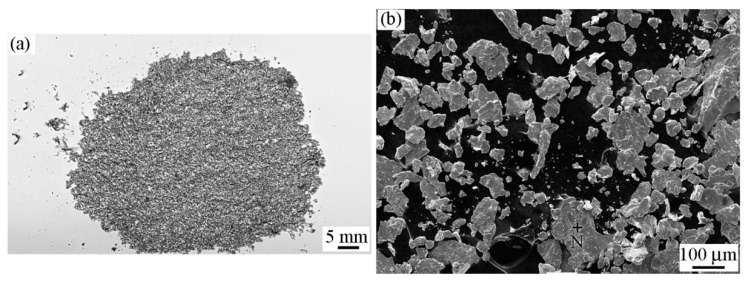
Morphology of the wear debris when the RD was used; *H* = 26.1 × 10^3^A/m. (**a**) Macro-photograph and (**b**) SEM image.

Figure 7a,b show a macro-photograph and SEM image of the wear debris collected in the friction process, respectively, when the RD was used. As shown, the wear debris was bright white and large. No agglomeration could be observed, as the wear debris was large in size. EDS results pertaining to zone N shown in Figure 7b are listed in Table 1. Oxygen content in this wear debris was much lower than that in the wear debris that was formed when the RD was not used during friction. This might suggest scarce oxidation of the wear debris when a RD is used.

### 4.2. Friction Process Using the RD

**Figure 8:**
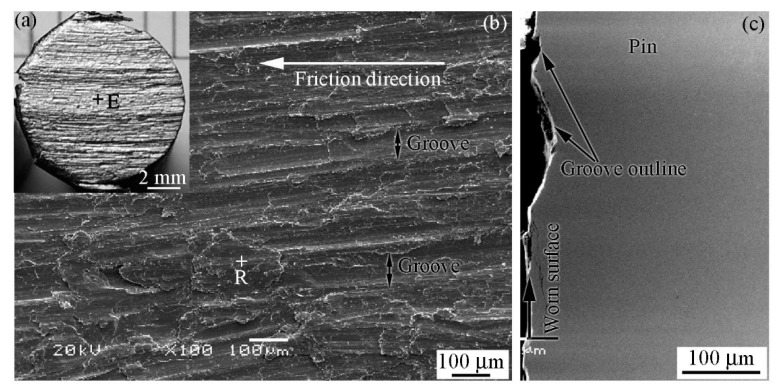
Features of the pin worn surface when the RD was used during the friction process; *H* = 26.1 × 10^3^ A/m. (**a**) Macro-photograph, (**b**) SEM image of the worn surface, and (**c**) SEM image of the cross section.

Figure 8a shows a macro-photograph of the worn surface of the pin specimen when the RD was used during the friction process. The contrast of the pin specimen worn surface was metallic bright and white. Figure 8b shows a SEM image of the worn surface; it was captured in zone E (Figure 8a). Figure 8c shows a cross sectional SEM image of the pin specimen perpendicular to the friction direction. As shown, grooves of various shapes and discontinuities were observed on the worn surface. Wear debris can be considered to be the main reason for groove formation, as the wear debris that is held between pin and disk specimens acts as an abrasive during friction [31]. In fact, grooves on the worn surface are an accumulated result of wear during friction. Therefore, grooves on the worn surface are related to the behavior and state of the wear debris during friction.

According to these observations, the group behavior of wear debris formed when the RD used can be divided into three stages, including formation-adsorption, movement accompanying the disk specimen, and removal, which can be used to qualitatively analyze their role during the friction process. 

**Figure 9:**
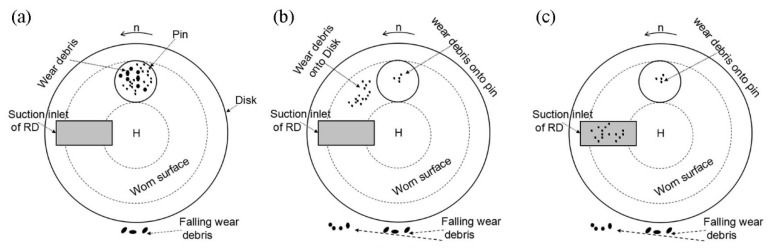
Behavior of wear debris when the RD was used. (**a**) Formation-absorption; (**b**) movement accompanying the disk; and, (**c**) removal.

Figure 9a shows a schematic diagram of wear debris formation-adsorption. In the initial stage of friction, micro-bulges on the surface of the specimen cause damage in the local zone, after which wear debris containing particles of different sizes is produced due to friction forces between the couple. Depending on its subsequent movement, the wear debris formed can generally encounter three situations. First, when the size of the wear debris is larger than the gap between the couple, the debris is held in the friction area by the pin and disk specimens and it leads to a backward movement of the pin specimen. Wear debris as abrasive grains results in the formation of grooves on the surface of the specimen during the relative movement of the pin and disk specimens. Secondly, when the size of the wear debris formed is slightly smaller than the gap between the couple, it directly falls off from the friction area under the action of gravity. Thirdly, when the wear debris formed is so small that its magnetic attractive force is greater than gravity, it is adsorbed on the worn surface of the pin and disk specimen. Parts of the wear debris that absorbed on the worn surface of the pin specimen might fall off, followed by the formation of larger wear debris during the next friction cycle. Further, other parts still remain in the friction area. Therefore, the wear debris adsorbed on the worn surface of the pin specimen always undergoes a dynamic process of new and old state replacement throughout the friction process.

Figure 9b shows a schematic diagram of wear-debris movement accompanying the disk specimen. The wear debris absorbed on the worn surface of the disk specimen moves out of the friction area when the disk specimen undergoes rotation. As described previously, the magnetic attractive force on the wear debris gradually decreases with an increase in the rotating angle of the disk specimen. Therefore, a part of the wear debris might fall off from the worn surface of the disk specimen. Other smaller debris particles are still absorbed on the worn surface and they continue to move with the disk specimen.

Figure 9c shows a schematic diagram of the wear-debris removal. The wear debris adsorbed on the worn surface of the disk specimen is sucked off by the RD when it is rotated to the suction inlet of the RD. Therefore, little wear debris exists on the worn surface after the disk specimen leaves the suction inlet of the RD.

**Figure 10:**
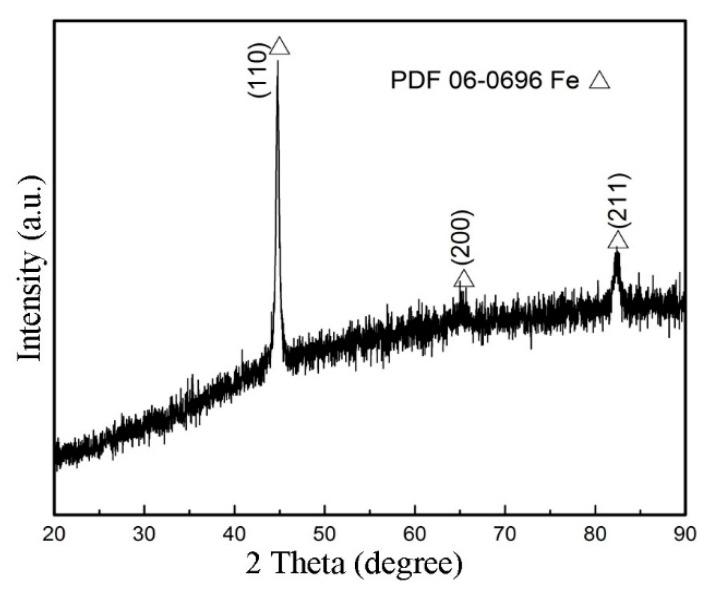
XRD pattern at the pin worn surface after using the RD.

It is worth noting that the three stages experienced by the wear debris, as mentioned earlier, are not isolated but interlaced and run throughout the friction process. The features of the worn surfaces of the pin specimens shown in Figure 8 are cumulative results of repeated actions in the three stages. The EDS results in zone R in Figure 8b indicate that the Fe content was 86.82% (at.%) and oxygen content was 13.18% (at.%). Figure 10 shows the XRD result at the worn surface of the pin specimen. As shown, only iron was detected on the worn surface. This indicates that no oxide layer was produced on the worn surface of the pin specimen during friction when the RD was used. A similar result was obtained on the worn surface of the disk. This is also the reason for the worn surface being bright white in this friction condition.

The specimens were constantly worn out during the friction process due to continuous wear-debris formation, movement of the disk specimen, and removal of the wear debris, which resulted in changes in the displacement of the pin specimen. In other words, changes in the pin specimen displacement can describe the dynamic wear of the pin specimen and some other information during friction.

**Figure 11:**
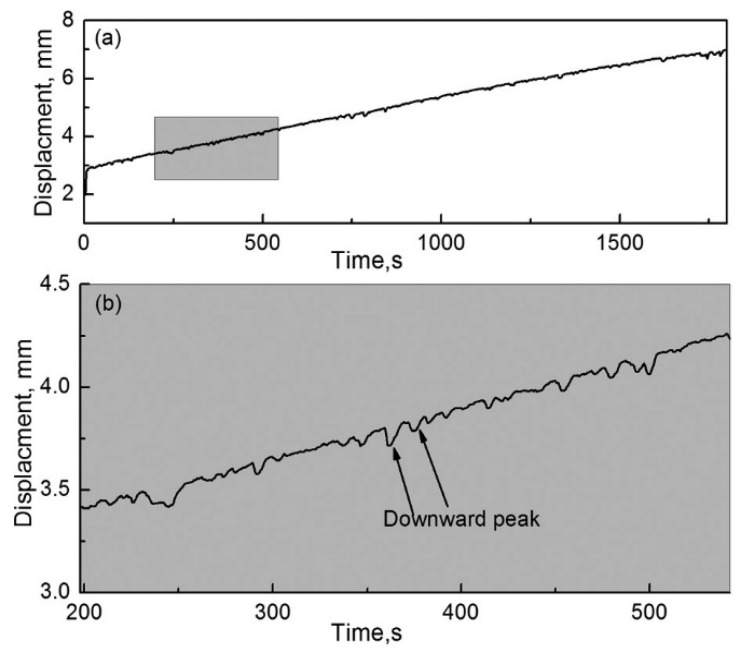
Pin-displacement evolution during friction when the RD was used; *H* = 26.1 × 10^3^ A/m. (**a**) Complete drawing and (**b**) local enlarged drawing of the shadowed region in (**a**).

Figure 11a,b show the displacement evolution of the pin specimen with the use of the RD and its local enlarged drawing, respectively. As shown in Figure 11a, displacement of the pin specimen rapidly increases with an increase in friction time. This indicates that the wear of the pin specimen rapidly increases as friction progresses. In addition, the slope of pin specimen displacement is constant on the whole, which reveals that the wear mechanism of the couple is the same throughout the friction process. Therefore, the results and features of the worn surfaces that are described above reveal that abrasive wear occurs between the couple when the RD was used.

As shown in Figure 11b, some downward peaks of various sizes are distributed on the displacement curve of the pin, which indicates that the pin specimen moves backward and the gap between the couple is enlarged at varying degrees. This is because a large amount of wear debris is produced during the friction process, inducing backward movement of the pin specimen. As the friction process continues, the large wear debris formed falls off from the friction zone, as mentioned earlier, and the pin displacement is reset.

### 4.3. Friction without Use of the RD

**Figure 12:**
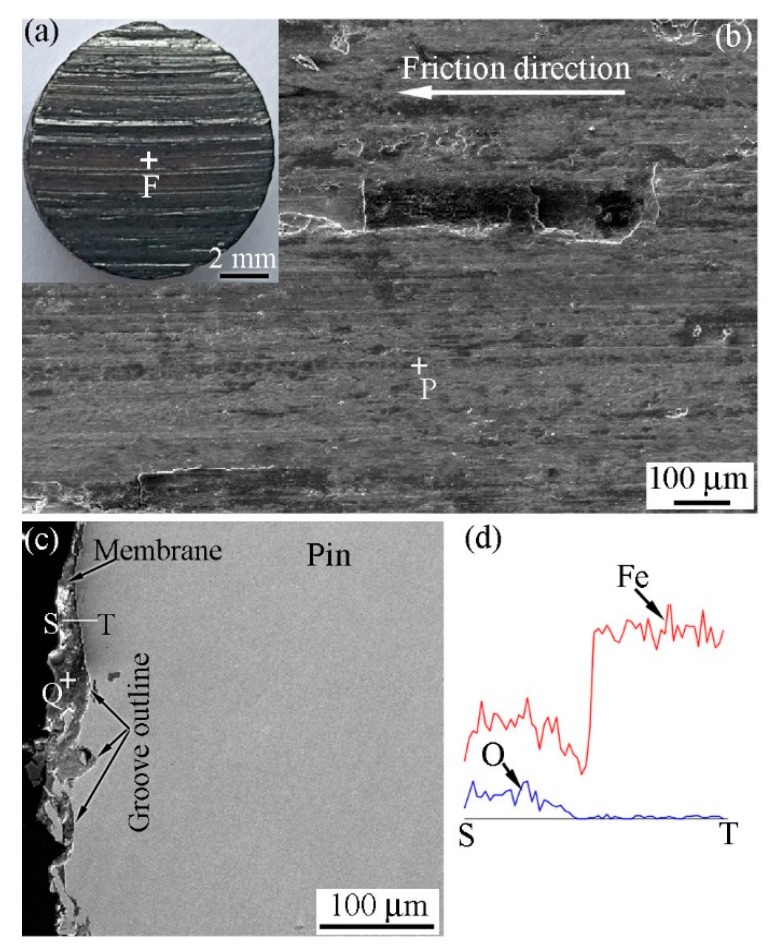
Features of the worn pin surface when the RD was not used; *H* = 26.1 × 10^3^ A/m. (**a**) Macro-photograph; (**b**) SEM image of the worn surface; (**c**) SEM image of the cross section; and, (**d**) energy spectrum analyzer (EDS) results along the ST line.

Figure 12a shows a macro-photograph of the worn surface of the pin specimen that formed when the RD was not used during the friction process. The contrast of the pin specimen worn surface was brown and black. Figure 12b shows a SEM image of the worn surface taken from the F zone in Figure 12a. As shown, a membrane layer and traces of grooves could be observed on the worn surface of the pin specimen. Similar phenomena were observed on the worn surface of the disk specimen.

**Figure 13:**
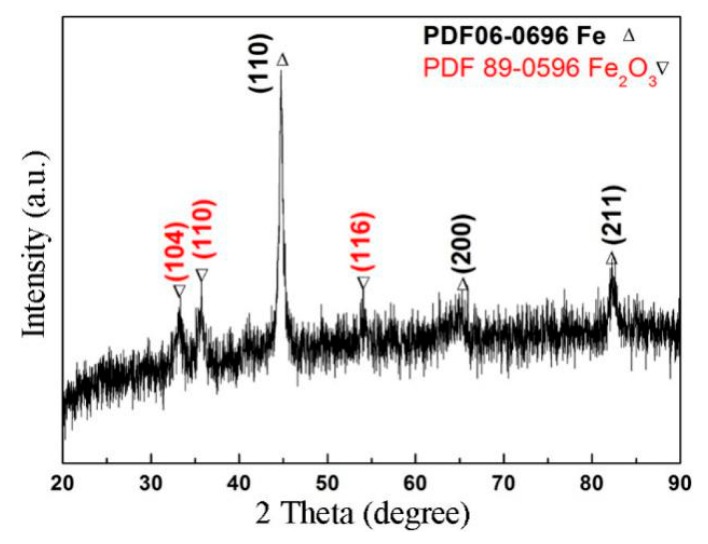
XRD pattern at the worn pin surface when the RD was not used.

Figure 12c shows a cross sectional SEM image of the pin specimen perpendicular to the friction direction. As shown, a membrane layer was observed at the worn end of the pin specimen. The membrane thickness was uneven across the worn surface. Its thickness on the grooves was higher when compared to other areas. Figure 12d shows the EDS results that were detected along the ST line in Figure 12c. On the membrane side, the content of O was higher, while that of Fe was lower when compared to their values on the pin specimen side. This indicates that the membrane is an oxide layer. It can also be seen from these results that Fe and O diffused through each other at the interface between the membrane and pin itself. Therefore, metallurgical bonding might occur at the interface between the membrane and pin. EDS analysis results at the locations P and Q that are shown in Figure 12 are listed in Table 2. The results show that the composition of the membrane is close to that of Fe_2_O_3_. Figure 13 shows the XRD pattern at the worn surface of the pin specimen. As illustrated, Fe_2_O_3_ and Fe could be detected. The detection of Fe_2_O_3_ indicates that the membrane contains Fe_2_O_3_, whereas the presence of Fe suggests that the Fe_2_O_3_ membrane is a discontinuous layer on the worn surface of the pin specimen. A similar result was also detected on the worn surface of the disk specimen. Further, this is also the reason why the worn surface is brown and black. 

**Figure 14:**
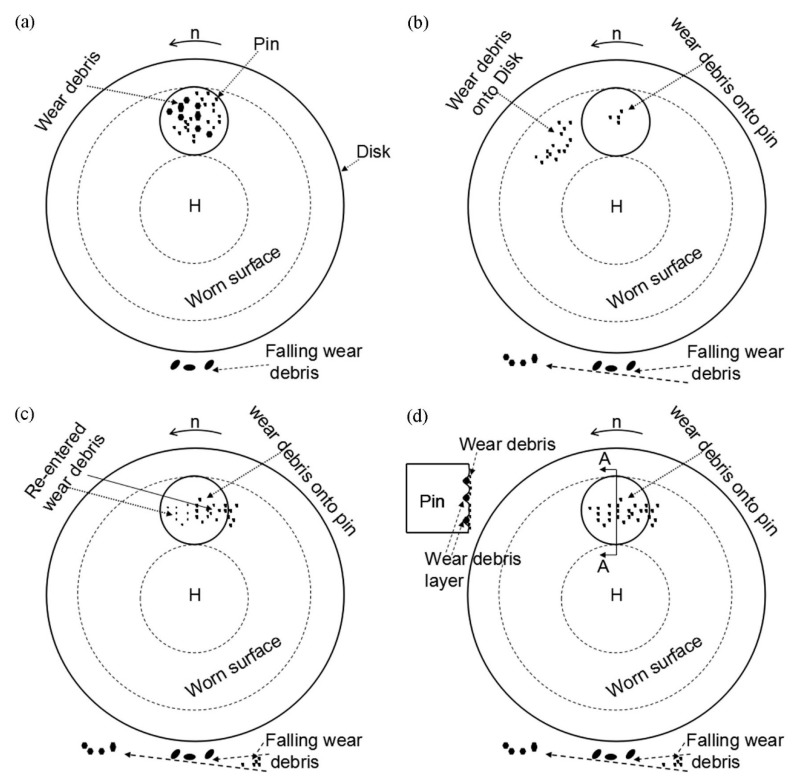
Behavior of the wear debris when the RD was not used. (**a**) Formation-absorption; (**b**) movement accompanying the disk; (**c**) grinding; and, (**d**) bonding.

According to the above-described observations, the group behavior of wear debris that formed when the RD was not used can be divided into four stages, including formation-adsorption, movement accompanying the disk specimen, grinding, and bonding, which can be used to qualitatively analyze its role during the friction process. Figure 14a,b show schematic diagrams of wear debris formation-adsorption and movement accompanying the disk specimen, respectively. The group behavior of wear debris in the two stages is the same as those when the RD was used.

Figure 14c shows a schematic diagram of wear-debris grinding. Parts of the wear debris adsorbed on the worn surface of the disk specimen can re-enter the friction area and participate in the friction process multiple times. The wear debris rubbed against the couple and probably broke into pieces under the action of a pressure perpendicular to the worn surface and friction force during the friction process, which refined the debris [31]. This is also the reason for refinement in the wear debris (Figure 5) under the conditions that were used in this study. As the worn surface of the disk specimen is in the vertical position, magnetic attraction force between the wear debris and the disk specimen is necessary for the re-entry of wear debris into the friction area. The influence of wear-debris grinding in this stage is similar to that of magnetic polishing [32,33].

Paramagnetic oxygen molecules can be attracted by the applied magnetic field, which increases the partial pressure of oxygen in the friction area and near the worn path of the disk [2]. In general, the extent of oxidation of metallic materials increases with an increase in the partial pressure of oxygen and specific surface area. Because repeated grinding of the wear debris results in an increase in the specific surface area, the oxidation of the wear debris occurred during the friction process when the RD was not used; in fact, it can even burn occasionally. This is also the reason for the large amount of oxygen that was detected in the wear debris.

Figure 14d shows a schematic diagram of bonding. The wear debris adsorbed on the worn surfaces of the pin and disk specimens at the friction area is pressed into the grooves during friction. Bonding occurs between the wear debris and specimen itself in the grooves under the action of a normal pressure. Later, more wear debris continues to accumulate on the wear debris bonded to the specimen itself. The process is repeated, and thus, a wear-debris layer is formed on the grooves, as shown in Figure 12c. 

According to the solid-state bonding theory, the metallurgical bonding process between two materials is as follows. The material at the interface undergoes plastic deformation under the action of an applied force. Bonding is produced via the mutual diffusion of atoms. Thus, the key factors for bonding include the applied force, plastic deformation, and diffusion [34]. 

This theory can also be applied to explain the bonding between the wear-debris layer and the specimen itself. Firstly, the applied force is obtained. Different and discontinuous grooves can impede the movement of wear debris along the groove during friction. Agglomeration of wear debris can cause the force generated due to holding the debris between the pin and disk specimens to be applied on the wear debris in the grooves on the worn surface. Secondly, plastic deformation occurs. Fine wear debris is compressed and induces plastic deformation. This can also result in plastic deformation in the specimen surface where it is in contact with the wear debris. These conditions provide the necessary environment for atomic diffusion. Thirdly, atom diffusion occurs at the contact area between the wear debris and the specimen. A part of the surface of the specimen presents the blue and yellow phenomenon during wear-debris grinding, as shown in Figure 6. This reveals that oxidation occurs in the partial zone on the specimen surface, where it is grinded by the wear debris. Thus, Fe and O diffused through each other at the interface between the wear debris and pin. This provides suitable conditions for the bonding and formation of a common crystal lattice between the wear debris and pin.

In summary, metallurgical bonding occurred between the wear debris and the pin itself. Meanwhile, bonding also occurred in the agglomerated wear debris, as described earlier. As result, a wear-debris layer was formed on the groove of the worn surface.

After the formation of a wear-debris layer on the worn surface during the friction process, the behaviors of the wear debris also makes a difference from that mentioned above. Because the wear-debris layer separates the pin from the disk specimen, the formation of wear debris in this stage can be attributed to the wear product of the wear-debris layer bonded to the worn surface. When the grooves are filled with wear debris, the roughness of the worn surface reduces and the actual contact area between the couple grows larger. This weakens the concentration effect of the magnetic field at the contact point on the worn surface and it induces a uniform magnetic-field distribution [21]. 

The four stages of group behavior of the wear debris (formation-adsorption, movement accompanying the disk specimen, grinding, and bonding) are not isolated but interlaced and run throughout the friction process. The features of the worn surfaces of the pin and disk specimens are cumulative results of the repeated action of these four stages. In the end, wear-debris layer formation and destruction on the worn surface by friction and wear reach a dynamic balance. During friction, bonding between the wear-debris layer and the worn surface might have a certain impact on the friction and wear of the couple.

**Figure 15:**
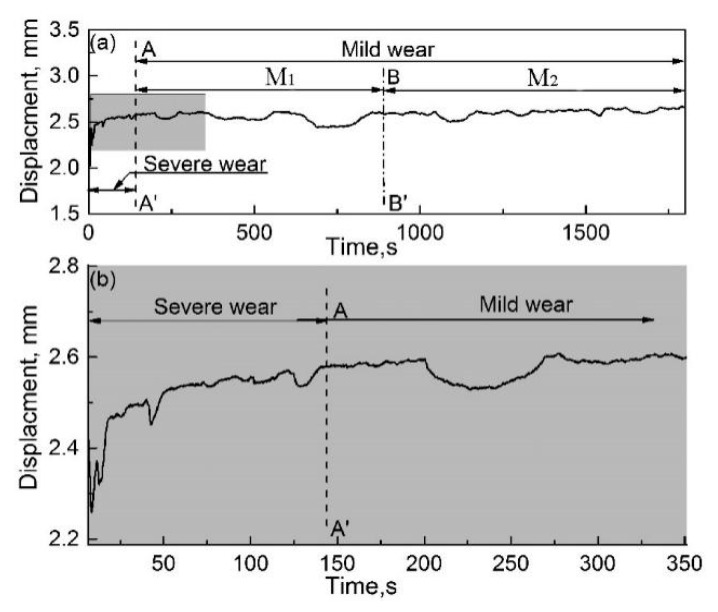
Pin-displacement evolution during friction when the RD was not used; *H* = 26.1 × 10^3^ A/m. (**a**) Complete drawing and (**b**) local enlarged drawing of the shadowed region in (**a**).

Figure 15a,b show the displacement evolution of the pin specimen with respect to an increase in the friction time when the RD was not used and an enlarged local drawing, respectively. As shown in Figure 15a, the complete displacement can be divided into two stages of severe wear and mild wear according to the slope characteristics.

In the severe-wear stage, the displacement of the pin specimen rapidly increases in the initial stage of friction. At the beginning of friction, less and large wear debris existed and fine and agglomerated wear debris was not established in the friction region. Therefore, the direct contact area between the couple is large and abrasive wear comes into being between the couple, which results in severe wear and produces grooves on the worn surface. 

In the mild-wear stage, the growth rate of the pin specimen displacement decreases rapidly and its slope is close to zero. In comparison with the displacement curve of the pin specimen when the RD was used (Figure 11), no downward peak was observed in the displacement curve in the second stage, as shown in Figure 15b, which indicates that no large wear debris was formed during this period. Moreover, the mild-wear stage can be divided into M_1_ and M_2_ stages according to its fluctuations. In M_1_, fluctuations in the displacement curve of the pin specimen are large. The agglomeration of wear debris between the couple made the pin move backwards and the wear debris fell off due to a decrease in the magnetic attraction force with an increase in displacement; subsequently, the pin moved forward. This is the reason for the value of pin displacement decreasing first and then increases when the friction time varies from 200 to 275 s. This might be due to the following reasons. On the one hand, the fine wear debris gradually accumulates and grows in number or quantity between the pin and disc specimens, owing to the magnetic attraction force. This can result in the moving backward of the pin specimens and then the decrease of its value. On the other hand, when the wear debris accumulates to a certain extent, the magnetic attraction is not enough to hold them between the pin and disk specimens and then the wear debris falls off from the gap. This might lead to the moving forward of the pin specimens and the increase of its value. With an increase in friction time, agglomerated and fine wear debris formed between the couple, eliminating direct contact between the pin and disk specimens. Because of the refinement of wear debris, the grinding process takes place in the M_1_ section, which resulted in the mild wear of the specimen.

In M_2_, the fluctuations in the displacement curve of the pin specimen are smaller. This is because wear-debris agglomeration became lesser due to a weakening of the concentration effect of the magnetic field at the contact point on the worn surface. When a wear-debris layer is formed on the specimens and a dynamic balance between its formation and destruction is reached, the friction couple is separated by this layer. Wear was mainly shared responsibility for the layer on the worn surface of specimen rather than the specimen itself, which contributes to mild wear (oxidation wear).

In other words, the transition from severe wear to mild wear occurred under these conditions when the friction time was ~140 s. Transition from severe wear to mild wear can also be observed even in the absence of a magnetic field [35,36]. Although no magnetic field is used, wear debris can remain on the worn surface during friction, because the disk specimen is installed in a horizontal position [35,36]. This might indicate that wear debris is a key factor in determining the wear of a specimen.

**Figure 16:**
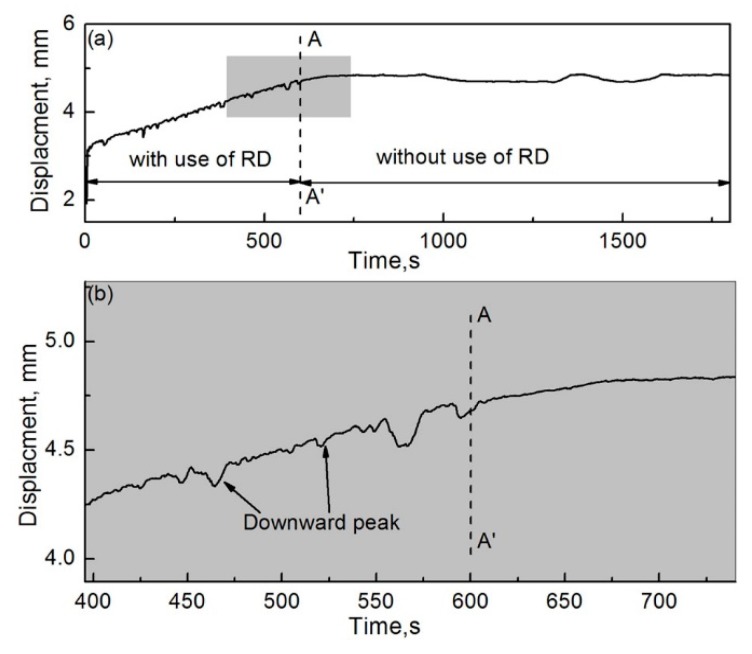
Pin-displacement evolution during segmented friction testing; *H* = 26.1 × 10^3^ A/m. (**a**) Complete drawing and (**b**) local enlarged drawing of the shadowed region in (**a**).

In order to further analyze the effect of wear debris on the wear properties of the friction couple, a segmented friction test was conducted. In the first stage (0–600 s), the RD was used. In the second stage (600–1800 s), the RD was not used. Figure 16a,b show the displacement evolution of the pin specimen with an increase in friction time and its local enlarged drawing, respectively. As shown in Figure 16a, the displacement of the pin specimen rapidly increased with an increase in friction time in the first stage. This indicates that severe wear occurred between the friction couple. In addition, the displacement growth rate of the pin specimen decreased rapidly, and its slope was close to zero when the friction time was greater than 600 s. Mild wear occurred between the friction couple in the second stage. As shown in Figure 16b, there are some downward peaks in the displacement curve in the first stage. These peaks vanished in the second stage. The reason for the generation and disappearance of these peaks was explained earlier. The test results can intuitively illustrate the effect of wear debris on the wear properties between the couple.

### 4.4. Dynamic Friction Coefficient

**Figure 17:**
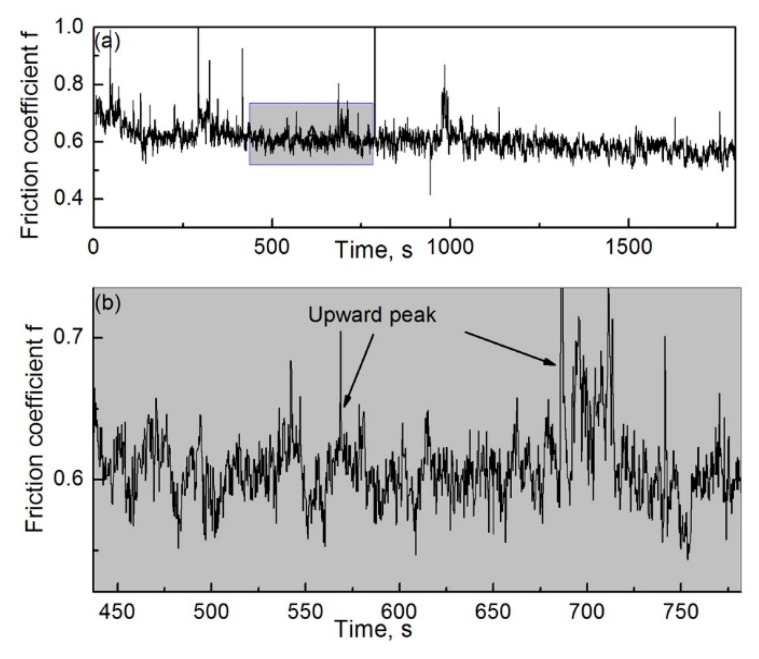
Friction–coefficient evolution during the friction process when the RD was used; *H* = 26.1 × 10^3^ A/m. (**a**) Complete drawing and (**b**) local enlarged drawing of the shadowed region in (**a**).

Figure 17a,b show the evolution of friction coefficient when the RD was used in the friction process and its local enlarged drawing, respectively. As shown in Figure 17a, the changing trend of the friction coefficient is consistent on the whole throughout the friction process. The curve of the friction coefficient showed a large fluctuation. This is because the behavior of the wear debris and the characteristics of the worn surface remained unchanged throughout the friction process, as mentioned earlier. As shown in Figure 17b, there occur several various upward peaks in the dynamic friction–coefficient curve. The wear debris was large when the RD was used in the friction process. The production of larger wear debris might consume more power, which results in an increase in the instantaneous friction coefficient.

**Figure 18:**
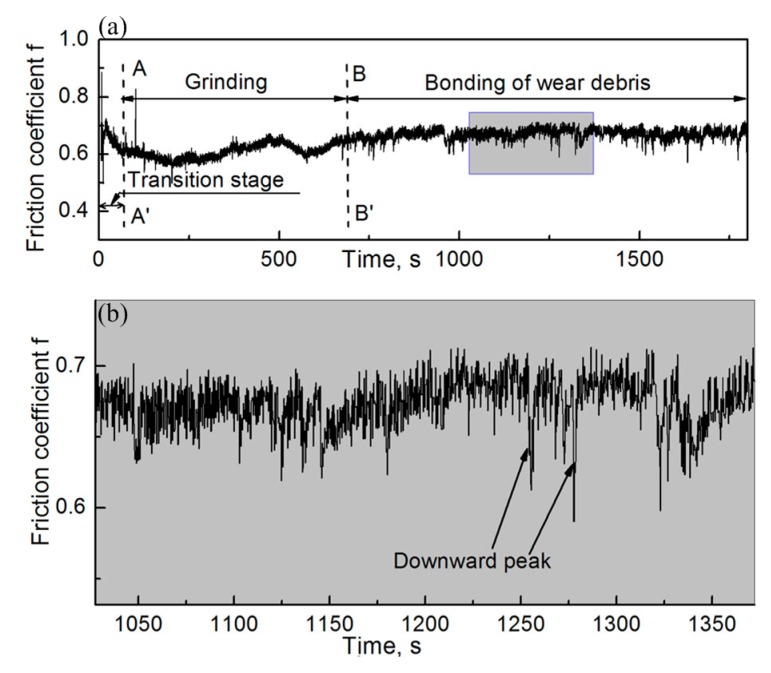
Friction–coefficient evolution during the friction process without using the RD; *H* = 26.1 × 10^3^ A/m. (**a**) Complete drawing and (**b**) local enlarged drawing of the shadowed region in (**a**).

Figure 18a,b show the evolution of friction coefficient when the RD was not used in the friction process and its local enlarged drawing, respectively. As shown in Figure 18a, the evolution of friction coefficient with respect to friction time can be divided into three stages, including the transition stage, grinding stage, and bonding stage, according to the characteristics of the friction process.

In the transition stage, wear debris gradually formed between the couple. The wear debris was lesser and larger at the beginning of the friction process and it may produce grooves on the worn surface. Therefore, the friction coefficient is large in this stage. In the grinding stage, a large quantity of fine and agglomerated wear debris was formed between the couple. The pin and disk specimens were separated from each other by this kind of wear debris. The wear debris acted as a solid lubricant between the specimens. Therefore, the friction coefficient at this stage decreased. In the bonding stage, the friction coefficient increased and was later stabilized. The wear-debris layer was mainly responsible for friction in this stage. Oxide in the wear-debris layer is harder than the specimen material itself and it is difficult to produce plastic deformation [31], which might result in an increase in the friction coefficient. 

It can be seen that there are some downward peaks in the dynamic friction-coefficient curve shown in Figure 18b. When the wear-debris layer responsible for friction was destroyed, the wear-debris layer located at the lower zone of the worn surface of the couple started to take part in the rubbing process. The interval time of the process might result in a decrease in the instantaneous friction coefficient and it produces a downward peak in the friction–coefficient curve. On the other hand, the worn surface of the specimen became flat and hard when the wear-debris layer was produced in the grooves. When the wear debris layer is destroyed during friction, it might break into pieces and roll over between the pin and disk specimens on the worn surface [37], thus reducing the instantaneous coefficient of friction. Later, when it detached from the friction area due to disk movement, the friction coefficient went back to its original state. 

In summary, it is obvious that the behavior of wear debris has a significant effect on the dynamic friction coefficient during the friction process.

### 4.5. Wear Coefficient and Anti-Wear Degree

**Figure 19:**
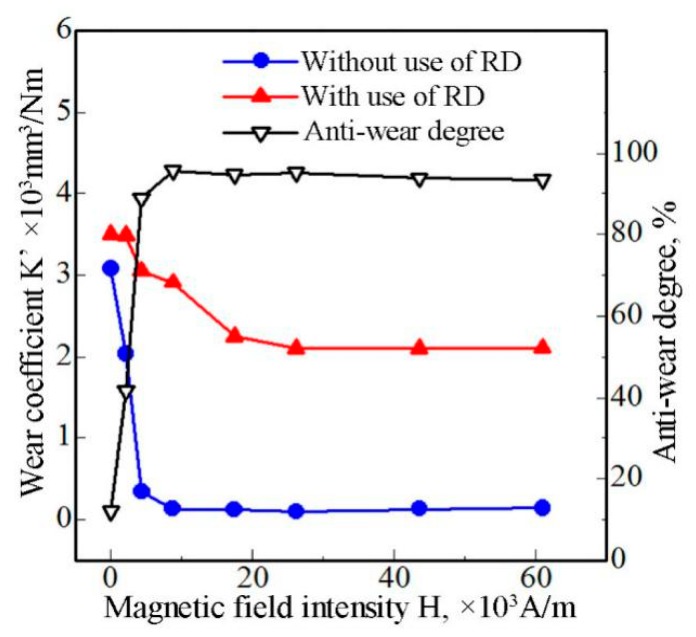
Wear coefficient and anti-wear degree.

Figure 19 shows the wear coefficients at different magnetic-field intensities, both when the RD was used and not used, and the anti-wear degree influenced by the wear debris.

First of all, the wear coefficients of the pin specimen (without RD and with RD) gradually decreased and reached a stable state with an increase in the magnetic-field intensity. The growth of the wear debris absorbed on the worn surface of the specimen increased with an increase in the magnetic-field intensity. The more the wear debris in the friction area, the easier it is for refinement and agglomeration of the wear debris to occur. When the wear debris in the friction area is fine and agglomerated, mild wear occurs between the couple as mentioned earlier. Therefore, the wear coefficients of the pin specimen decreased when the magnetic-field intensity increased. On the other hand, when the magnetic-field intensity reached a specific value, the wear debris absorbed on the surface of the specimen reaches the maximum corresponding to the stability value of wear coefficient. This is why the wear coefficients of the pin specimen gradually decreased and they reach a stable state with an increase in magnetic-field intensity.

Secondly, the wear coefficient of the pin specimen (using the RD) is greater than that obtained without using the RD at the same magnetic-field intensity. As described earlier during the analysis of the behavior and effect of wear debris, direct contact was dominant and adhesive wear occurred when the RD was used. This can result in severe wear. Conversely, the pin and disk specimens were separated by the wear debris and/or wear-debris layer at different stages of the friction process when the RD was not used. The fine and agglomerated wear debris and/or wear-debris layer shared most of the wear, and the wear of the specimen itself reduced significantly during the friction process. Therefore, the wear coefficient of the pin specimen, when the RD was used, is greater than that when no RD was used at the same magnetic-field intensity.

At the same magnetic-field intensity, the only difference between the two cases (with and without RD) is whether fine and agglomerated wear debris and/or wear-debris layer is formed or not during the friction process. It can be concluded that the wear performance of the specimen is significantly affected by the wear debris in the friction area. The difference in the values of the wear coefficient between the two cases at the same magnetic-field intensity might be generally considered as the reduction wear effect of the pin specimen that is caused by the wear debris. To put it succinctly, the wear debris and its characteristics in the friction area exert a remarkable anti-wear function. 

The anti-wear degree is used to quantitatively express the reduction wear effect of the wear debris; it is expressed as $△K=(K_{2}-K_{1})/K_{2}\times 100\%$. Here, *K*_1_ is the wear coefficient without using the RD and *K*_2_ is the wear coefficient when the RD was used. The calculated results are plotted in Figure 19. It can be seen that the wear coefficient can be reduced by ~94.5% by the wear debris.

## 5. Conclusions

Based on the experimental results that were obtained in this study, the behavior and characteristics of wear debris in the friction area have an influence on the tribological properties of medium carbon steel with a magnetic field. The following conclusions could be drawn.

(1) The presence of wear debris of fine, aggregation and oxidation in friction area can result in the formation of a wear-debris layer on the worn surface of the specimen, which can cause a transformation of wear mode from severe to mild.

(2) When the wear debris is removed, diverse and discontinuous grooves can be formed on the worn surface of the specimen and severe wear continues throughout the friction process.

(3) Re-entering of wear debris into the friction area is essential for the formation of a wear-debris layer that play an anti-wear role. The wear coefficient can be reduced by ~94.5% and the fluctuation of friction coefficient decreases during friction process.

## Figures and Tables

**Table 1:** Chemical components of the wear debris (at.%).

Measured Zone	Fe	O
M zone	72.35	27.65
N zone	94.60	5.40

**Table 2:** Chemical components of the worn surface (at.%).

Test Location	Fe	O
P	46.59	53.41
Q	43.99	56.01
